# Genetic variant in *IL-33* is associated with idiopathic recurrent miscarriage in Chinese Han population

**DOI:** 10.1038/srep23806

**Published:** 2016-03-30

**Authors:** Jun Yue, Yu Tong, Lan Xie, Tao Ma, Jiyun Yang

**Affiliations:** 1 Department of Gynecology & Obstetrics, Hospital of the University of Electronic Science and Technology of China and Sichuan Provincial People’s Hospital, Chengdu, China; 2 Prenatal Diagnosis Center, Hospital of the University of Electronic Science and Technology of China and Sichuan Provincial People’s Hospital, Chengdu, China; 3 Laboratory of Early Developmental and Injuries, West China Institute of Woman and Children’s Health, West China Second University Hospital, Sichuan University, Chengdu, China; 4 Key Laboratory of Gynecologic & Obstetric and Pediatric Diseases and Birth Defects of Ministry of Education, Chengdu, China; 5 Sichuan Provincial Key Laboratory for Human Disease Gene Study, Hospital of the University of Electronic Science and Technology of China and Sichuan Provincial People’s Hospital, Chengdu, China; 6 School of Medicine, University of Electronic Science and Technology of China, Chengdu, China

## Abstract

Recurrent miscarriage (RM) is the occurrence of repeated pregnancies that end in miscarriage of the fetus before 20 weeks of gestation. At least 50% of the RM patients are considered idiopathic. High IL-33 levels are critical in early pregnancy and impact the outcome of subsequent pregnancies. However, the association of polymorphisms of *IL-33* with idiopathic RM is still unclear. The present study was initiated to investigate whether *IL-33* polymorphisms are risk factors for idiopathic RM in Chinese Han population. Study subjects comprised of 321 cases and 384 controls. Five polymorphisms (rs10435816, rs16924159, rs16924171, rs1929992, rs1332290) in *IL-33* and serum IL-33 concentrations were assessed. rs16924159 variant exhibits significant association with RM in additive and recessive genetic model (additive model *P* = 0.015, recessive model *P* = 0.007). In contrast, rs10435816, rs16924171, rs1929992 and rs1332290 are not significantly associated with RM. Serum IL-33 levels are significantly lower in RM cases than in control (173.51 ± 94.12 versus. 200.97 ± 110.06 (pg/ml), *P* = 4.57 × 10^−4^). There are lower levels of serum IL-33 in rs16924159 homozygous mutant (AA) than homozygous wild-type (GG) in this study population, including cases and control groups (172.18 ± 103.01 versus. 205.82 ± 119.01 (pg/ml), *P* = 0.006). Reduced IL-33 levels and rs16924159 *IL-33* variant may contribute to the pathogenesis of idiopathic RM in Chinese Han population.

Recurrent miscarriage (RM) is the loss of three or more consecutive pregnancies before 20 weeks of gestation. RM affects 1–5% of women who attempt to bear children^1^. Various factors have been identified for miscarriage, including uterine anomaly, chromosomal abnormalities, endocrine dysfunction, thrombophilia, immune disorders, life style factors and maternal infections^1^. However, At least 50% of the RM patients have no deviations from any applied diagnostic test. The underlying causes remain unknown and are considered idiopathic. Idiopathic RM is characterized by multifactorial etiology. Although much work has been done to identify the cause of idiopathic RM, its reason remains unknown^2^. There is growing evidence that RM has also genetic susceptibility^2^,^3^,^4^. In most conducted genetic association studies targeting RM, the most frequently addressed genes in the context of RM are associated with immunotolerance, inflammation and changes of maternal metabolism and blood coagulation^3^.

Successful pregnancy depends on the cytokine environment, which can either be protective or harmful to the conceptus^5^. The balance of pro-inflammatory and anti-inflammatory cytokines was suggested to be critical for successful pregnancy^6^. Maternal immunological tolerance of the allogenic fetus in pregnancy is linked with imbalance between T regulatory cells (Tregs) and T helper 17 cells (Th17)^7^,^8^. There is an elevation of Treg subset in the normal human pregnancy. However, a decrease in the number of Tregs is associated with miscarriage^9^,^10^. In contrast to the Tregs cells, the proportion of Th17 cells was significantly higher in patients with RM than in normal pregnant^11^,^12^,^13^.

IL-33 is a member of the IL-1 family. Members of this family are known to play important roles in host defense, immune regulation, neuronal injury and inflammation^14^. IL-33 is mainly expressed in epithelial, endothelial cells, activated Th2 cells and mast cells^14^,^15^,^16^,^17^. IL-33 appears to be a cytokine with dual function, performing both as a traditional cytokine through activation of the IL-33/ST2 pathway and as a nuclear factor involved in transcriptional repression and regulation of genes^18^,^19^. IL-33 has been reported to be the specific ligand for suppression of tumorigenicity 2(ST2), which is a member of the IL-1 receptor superfamily. IL-33 significantly stimulated the production of inflammatory cytokines, such as IL-1β, TNF-α, IL-8 and IL-6^20^. IL-33 enhances Tregs cells differentiation. Furthermore, it provides a necessary signal for Tregs cells accumulation and maintenance in inflamed tissues^13^,^21^.

Recent research shows that IL-33 expression was found in endothelial and smooth muscle cells in the placenta, chorioamniotic membranes, and umbilical cord^22^. Human endometrial stromal cells (HESCs) rapidly release IL-33. *IL-33* knockdown in undifferentiated HESCs abrogate this pro-inflammatory decidual response^23^. Further, sequential activation of the IL-33/ST2L/sST2 axis was disordered in decidualizing HESCs from women with recurrent pregnancy loss. Signals from HESC cultures prolonged the implantation window but also caused subsequent pregnancy failure in mice^23^. Serum IL-33 levels of normal pregnancies were highly variable in first eleven weeks of early pregnancy, with no statistical differences identified between each gestational week^24^. However, there is a significant increase in serum IL-33 levels from patients who went on to abortion compared with normal control pregnancies^24^. Above data suggest that dysregulated levels of serum IL-33 indicate miscarriage in live early pregnancies.

However, the association of polymorphisms of IL-33 with idiopathic RM is still unclear. The present study was initiated to evaluate association of the *IL-33* gene polymorphisms with idiopathic RM in Chinese Han population. Five polymorphisms (rs10435816, rs16924159, rs16924171, rs1929992, rs1332290) in *IL-33* gene were selected for genotyping, and then we analyzed the association between the five SNPs and the risk of idiopathic RM.

## Materials and Methods

### Subjects

This study has been carried out in accordance with The Code of Ethics of the World Medical Association (Declaration of Helsinki) for experiments involving humans. The Institutional Review Boards of the Sichuan Provincial People’s Hospital and West China Second University Hospital, Sichuan University approved this study. All subjects provided informed consent before participating in the study.

Cases comprised 321 women from Sichuan Provincial People’s Hospital and West China Second University Hospital between January 2011 and June 2013, who had experienced three or more pregnancy losses with the same partner, and which had occurred from between the beginning of pregnancy to the 20th week of gestation; gestational age was calculated as the time between the first day of the last normal menstrual period and the first signs of pregnancy loss. The work-up included endometrial biopsies for evaluating luteal phase defects;pelvic ultrasound scans to assess ovarian morphology and the uterine cavity; hysteroscopy to evaluate uterine anatomic abnormalities; karyotyping of peripheral blood to evaluate chromosomal aberrations in both patient and partner. These procedures were performed on all patients. In addition, trisomy and triploidy of fetal tissues and were evaluated using fluorescence *in situ* hybridization (FISH). Data on FISH of fetal tissues were available for 266 (82.87%) patients.

Exclusion criteria included older age (40 years or older at first miscarriage), parental and fetal chromosomal aberrations, Rh incompatibility, anatomical abnormalities, preclinical miscarriages, endocrine disorders (including diabetes), liver function abnormalities, and abnormal thyroid function, thyroid antibodies, hyperprolactinemia prior to luteal phase defects, and fetomaternal alloimmune thrombocytopenia, infections (toxoplasmosis, human cytomegalovirus, rubella, human immunodeficiency virus, Group B streptococci, Chlamydia trachomatis, hepatitis B and C and bacterial vaginosis). Patients were also excluded if they reported systemic autoimmune disease, such as anti-phospholipid syndrome, systemic lupus erythematosus, multiple sclerosis, rheumatoid arthritis.

Control subjects composed of 384 women who were recruited from Sichuan Provincial People’s Hospital and West China Second University Hospital between January 2011 and June 2013. Age and ethnical group were matched with patients. All subjects had at least one live birth and no miscarriages, and did not have a family history of RM.

### *IL-33* genotyping

Blood samples were taken from all participants in EDTA containing tube for total genomic DNA extraction. Total genomic DNA was extracted by silica columns isolation kits (Tiangen BioTech CO.LTD, Beijing China).

Genotype data of *IL-33* for the chromosomal region 9:6215149–6257983 were download from the HapMap website (CHB database, Hapmap Data Rel 27 Phase II III, Feb09, on NCBI B36 assembly, dbSNP b126). In total, 37 SNPs with a frequency >0.10 were identified in CHB (Han Chinese in Beijing). The criteria for all the referenced SNPs was that minor allele frequency (MAF) set at >10% and r^2^ > 0.8. The five tag-SNPs (rs10435816, rs16924159, rs16924171, rs1929992, rs1332290) were selected by running the tagger program in Haploview 4.2. These five SNPs in 5 tests capture 37 of 37 (100%) alleles with a mean r^2^ of 0.957. rs10435816, rs16924159 and rs16924171 are located in the 5′ upstream region of IL-33 (NM_001199640.1), rs1929992 and rs1332290 are located in the intron region of IL-33 (NM_001199640.1). *IL-33* genotyping was performed using real time PCR assays (TaqMan probe). TaqMan® SNP Genotyping Assay kit were from Applied Biosystems. Cat. C__30088929_10 (rs10435816), C__34291196_10 (rs16924159), C__34291189_10 (rs16924171), C__11716827_10 (rs1929992), C___2762144_10 (rs1332290). Replicate blinded quality control samples were included to assess the reproducibility of the genotyping procedure. The successful genotyping rate was 99.0%. In brief, 24 individuals of cases and controls were randomly selected to sequence for confirmation of five SNPs genotyping. The confirmation rate was 100%.

### Serum IL-33 Measurements

Because pregnancy influences IL-33 levels, blood drawing criteria were used for patients and control women. This included interval (4–6 months) from the last event (miscarriage or delivery) and time of blood draw (8:00–10:00 AM). Blood samples were taken from all participants in plain tubes (no preservatives) for serum preparation. Serum was prepared by centrifugation of coagulated blood tubes at 2000 g for 10 min at room temperature, and was stored in 200 μl aliquots at −80 °C. Serum IL-33 were measured using a human IL-33 enzyme-linked immunosorbent assay (catalogue number ADI-900-201; Enzo Life Sciences., Shanghai China) according to the manufacturer’s instructions. Assay sensitivity was 1.7 pg/ml (range: 7.8–500 pg/ml). Samples were tested in triplicate at the same time in the same assay.

### Statistical analysis

Statistical analysis was performed by the software SPSS for Windows (version 13.0). Continuous variables were expressed as mean ± SD. Statistical differences were performed using t test or Mann–Whitney U test. Then differences in genotype distribution of the polymorphism between case and control subjects were compared by χ^2^ test. Three models were used for statistical analysis, including dominant model, the recessive genetic model and the additive model. Logistic regression analysis was used to evaluate the association between genotype of SNPs and idiopathic RM. Subsequently, Pearson χ^2^ statistics were calculated and *P* values were computed by comparing the statistic to a χ^2^ distribution with 1 or 2 degrees of freedom for the allelic and genotypic tests. *P* value of less than 0.05 was considered statistically significant. Gene variants were tested for Hardy-Weinberg equilibrium using Haploview version 4.2 (http://www.broad.mit.edu/mpg/haploview).

We used CaTS Power Calculator (www.sph.umich.edu/csg/abecasis/cats) to calculate the power to detect an association between *IL-33* variants and RM in our cohort. The parameters used were: 321 RM cases and 384 control women, genotypic relative risk for heterozygotes (1/2) and minor allele homozygote (2/2), and the MAF for RM cases and controls for the five tested SNPs, and assumed 5% population prevalence of RM. Assuming these parameters, this sample size provided 82%, 83%, 82%, 82% and 83% power for rs10435816, rs16924159, rs16924171, rs1929992 and rs1332290, respectively.

## Results

### The clinical characteristics of study participants

The clinical characteristics of patients and controls are listed in Table 1. There are no significant differences in mean age, smoking (%) and mean BMI (kg/m^2^) between cases and control (*P* > 0.05). While lower age at menarche, higher irregular menstrual history (%) and number of pregnancies were seen in the RM group. Although they did not constitute strong risk factors of RM, they were selected as the covariates that were controlled for in subsequent analyses.

### IL-33 genotype distribution

Genotype distribution of five (rs10435816, rs16924159, rs16924171, rs1929992 and rs1332290) SNPs are in Hardy-Weinberg equilibrium in cases and controls (*P* > 0.05).

Table 2 summarizes the distribution of *IL-33* genotypes between patients and control. Significant differences in the distribution of rs16924159 genotype are observed between patients and controls. rs16924159 variant exhibits significant association with RM in additive and recessive genetic model (additive model *P* = 0.015, recessive model *P* = 0.007). In contrast, rs10435816, rs16924171, rs1929992 and rs1332290 are not significantly associated with RM in any of three genetic models. As shown in Table 3, after adjustments for menarche age, irregular menstrual history and number of pregnancies by Binary Logistic Regression, rs16924159 showed significant association with RM in two genetic models (adjusted odds ratio (OR) = 2.037, 95% confidence interval (CI) = 1.135–3.656, *P* = 0.017 in an additive model; OR = 1.975, 95% CI = 1.161–3.357, *P* = 0.012 in a recessive model; OR = 1.926, 95% CI = 1.232–3.012, *P* = 0.014 in a homozygous model; OR = 1.199, 95% CI = 0.863–1.667, *P* = 0.299 in a heterozygous model).

### Mean serum IL-33 levels

**Figure 1:**
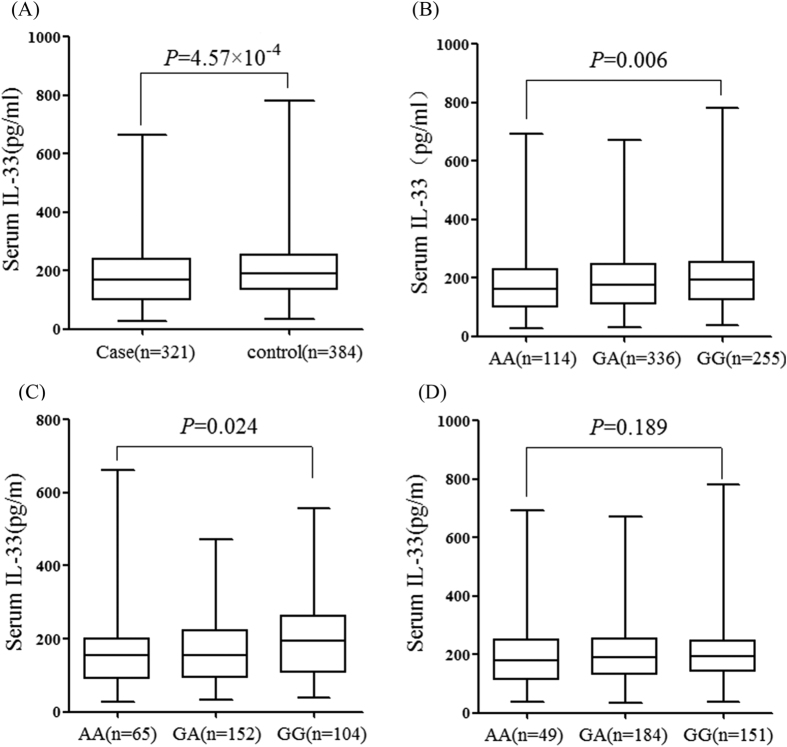
Comparison of serum IL-33 levels among *IL-33* genotypes. (**A**) Serum IL-33 concentrations in cases and controls. IL-33 levels were significantly lower in patients with RM than in controls. (**B**) Serum IL-33 levels according to rs16924159 genotypes among cases and controls. Women with AA genotype showed significantly lower IL-33 levels than GG genotypes. (**C**) Serum IL-33 levels according to rs16924159 genotypes among cases. Idiopathic RM patients with the AA genotype showed significantly lower serum IL-33 levels than those with GG genotypes. (**D**) Serum IL-33 levels according to rs16924159 genotypes among controls. No statistical difference of serum IL-33 levels was found between women with those with GG genotypes.

As shown in Fig. 1A, serum IL-33 levels are significantly lower in RM cases than in control (173.51 ± 94.12 versus 200.97 ± 110.06 pg/ml, *P* = 4.57 × 10^−4^). As shown in Fig. 1B, there are lower levels of serum IL-33 in rs16924159 homozygous mutant (AA) than homozygous wild-type (GG) in this study population, including cases and control groups (172.18 ± 103.01 versus 205.82 ± 119.01 pg/ml, *P* = 0.006). However, There is no significantly difference in homozygous wild-type (GG), compared with homozygous wild-type (GG) and heterozygous (A/G) genotype carriers (172.18 ± 103.01 versus 191.61 ± 103.91 pg/ml, *P* = 0.067).

Moreover, there is a significant decline in serum IL-33 levels according to rs16924159 genotypes in RM cohorts (AA versus GG, 160.57 ± 100.03 versus 196.90 ± 102.12 pg/ml, *P* = 0.024) (Fig. 1C). However, no significantly difference is observed in serum IL-33 levels according to rs16924159 genotypes among controls (AA versus GG, 187.57 ± 105.90 versus 211.96 ± 129.34 pg/ml, *P* = 0.189) (Fig. 1D).

### Haplotype analysis

Five-locus (*IL-33* rs10435816, rs16924159, rs16924171, rs1929992, rs1332290) *IL-33* haplotypes were constructed based on the prevalence of individual SNPs and linkage disequilibrium between them. Nine haplotypes were found to be common and were included in subsequent analysis. No haplotypes exhibits significant association with RM (Table 4).

## Discussion

The role of *IL-33* polymorphisms in idiopathic RM pathogenesis remains unknown. In this case–control study, we selected five tag SNPs in *IL-33* to investigate the association of *IL-33* polymorphisms with idiopathic RM. We found that a tag SNP rs16924159, located in the regulatory region of *IL-33*, was associated with idiopathic RM. The homozygous mutant (AA) genotype of rs16924159 is risk factor in idiopathic RM patients. This is the first study to assess the contribution of *IL-33* polymorphisms in hereditary susceptibility of idiopathic RM.

We found that serum IL-33 levels are significantly lower in idiopathic RM cases than in control. Recently, it has been reported that activation of the IL-33/ST2 pathway in human endometrial stromal cells (HESCs) is critical for a successful pregnancy^23^. Autocrine IL-33 signaling in HESCs facilitates embryo implantation in mice^23^. However, *IL-33* knockdown in decidualizing HESCs impairs embryo implantation^23^. Women experiencing with RM have deficit in Treg cell number and function, compared with normal pregnant women^25^. These studies suggest that high IL-33 levels are critical in early pregnancy and impact the outcome of subsequent pregnancies^23^. However, compared with normal control pregnancies, there is a significant increase in serum IL-33 levels at six weeks’ gestation in patients who destined to abort^24^. The biological significance of a rise of serum IL-33 levels is uncertain in pregnant woman destined to miscarry. As IL-33 is a crucial role in promoting Tregs cells differentiation and adaptation to the inflammatory environment. One possible explanation is that the pregnancy is failing due to sensitization of the maternal immune system, and that the rise is a compensatory response to attempting to rescue the pregnancy^24^. Although IL-33 plays an exact role in embryo-maternal interactions, especially during early stages of implantation, our data support the notion that miscarriage may be attributed, at least in part, to low level of IL-33 expression and secretion. In this study, serum IL-33 measurements were performed in non pregnant women. We did not compare serum IL-33 levels between pre-pregnancy with post-pregnancy in the same case. In later study, more experiments are also needed to elucidate the association of serum IL-33 levels between pre-pregnancy with post-pregnancy in idiopathic recurrent miscarriage patients.

Our data also indicate that there are lower levels of serum IL-33 in rs16924159 homozygous mutant (AA) than homozygous wild-type (GG) in this study population, including cases and control groups. Moreover, there is a progressive decline in serum IL-33 levels according to rs16924159 genotypes in RM cohorts. rs16924159 variant, located in the regulatory region of *IL-33*, was associated with reduced serum IL-33 levels. This may be linked with defective transcriptional processing of IL-33 mRNA.

To our knowledge, we first found that genetic variant in *IL-33* is associated with idiopathic RM, and the AA genotype of rs16924159 is associated with lower serum IL-33 levels. However, our study has some limitations. It was performed only in Chinese Han population. Additional more large-scale population studies from different ethnic backgrounds are needed to confirm the association of *IL-33* variant rs16924159 with RM. Functional experiments are also needed to elucidate the association.

In conclusion, our study demonstrated that a tag SNP rs16924159 was associated with idiopathic RM and serum IL-33 levels are significantly lower in idiopathic RM cases than in control. There are lower levels of serum IL-33 in rs16924159 homozygous mutant (AA) than homozygous wild-type (GG) in this study population. It may be concluded that *IL-33* acts as a risk factor in the pathogenesis of idiopathic RM.

## Additional Information

**How to cite this article**: Yue, J. *et al.* Genetic variant in *IL-33* is associated with idiopathic recurrent miscarriage in Chinese Han population. *Sci. Rep.* **6**, 23806; doi: 10.1038/srep23806 (2016).

## Figures and Tables

**Table 1:** Clinical Characteristics of the Study Population.

Characteristic	Cases	Controls	*P* value
Mean age (years)	31.35 ± 5.04	31.95 ± 4.32	0.089
Smoking (%)	25 (7.79)	33 (8.59)	0.584
Mean BMI (kg/m^2^)	21.31 ± 2.99	20.95 ± 3.06	0.366
Menarche(years)	12.88 ± 1.32	13.19 ± 1.18	0.013
Irregular menstrual history (%)	39 (12.15)	28 (7.29)	0.038
Number of pregnancies	4.91 ± 1.15	3.01 ± 1.28	<0.0001
Miscarriage	3.83 ± 0.725	0 ± 0	<0.0001

*P* versus controls; BMI, Body Mass Index.

**Table 2:** Association between SNPs and risk for idiopathic recurrent miscarriage in Chinese Han population.

SNP	Genotype	Case (n = 321)	Control (n = 384)	Additive		Dominant		Recessive	
				*P* value	Corrected *P*	*P* value	Corrected *P*	*P* value	Corrected *P*
rs10435816	AA	103 (0.321)	118 (0.307)						
	AG	142 (0.442)	174 (0.453)						
	GG	76 (0.237)	92 (0.240)	0.926	1	0.699	1	0.93	1
rs16924159	GG	104 (0.324)	151 (0.393)						
	GA	152 (0.474)	184 (0.479)						
	AA	65 (0.202)	49 (0.128)	0.006	0.03	0.057	0.285	0.007	0.035
rs16924171	AA	91 (0.283)	115 (0.299)						
	AT	159 (0.495)	183 (0.477)						
	TT	71 (0.221)	86 (0.224)	0.867	1	0.93	1	0.642	1
rs1929992	AA	67 (0.209)	98 (0.255)						
	AG	172 (0.536)	185 (0.482)						
	GG	82 (0.255)	101 (0.263)	0.264	1	0.819	1	0.147	0.735
rs1332290	AA	58 (0.181)	71 (0.185)						
	AC	148 (0.461)	189 (0.492)						
	CC	115 (0.358)	124 (0.323)	0.602		0.324		0.886	

*P* versus controls; *P* value by χ^2^ test. Corrected *P* = *P* value × 5 (the number of genotyped SNPs).

**Table 3:** Associated between rs16924159 variant with risk for idiopathic recurrent miscarriage after adjustment for Binary Logistic Regression.

	*P* value	Wald value	OR	95% CI
Additive	0.017	5.69	2.037	1.135–3.656
Dominant	0.288	1.127	1.244	0.831–1.862
Recessive	0.012	6.33	1.975	1.161–3.357
Homozygous	0.014	5.87	1.926	1.232–3.012
Heterozygous	0.299	1.036	1.199	0.863–1.667

Adjustments for menarche age, irregular menstrual history and number of pregnancies by Binary Logistic Regression; OR, odds ratio; CI, confidence interval.

**Table 4:** *IL-33* Haplotypes.

Haplotype^a^	Total^b^	Cases^b^	Control^b^	*P* value
GGTAC	0.376	0.389	0.36	0.2637
AGAGA	0.269	0.272	0.266	0.8109
AAAGA	0.187	0.182	0.192	0.6504
AATAC	0.034	0.031	0.037	0.5395
AAAAC	0.023	0.022	0.024	0.8519
GAAGA	0.017	0.017	0.017	0.9973
GGAAC	0.016	0.013	0.02	0.276
AGTAC	0.015	0.016	0.015	0.8443
AGAGC	0.012	0.011	0.013	0.7764

Note: ^a^*IL-33* rs10435816/rs16924159/rs16924171/rs1929992/rs1332290 haplotypes.

^b^Haplotype frequency.
